# International electronic health record-derived COVID-19 clinical course profiles: the 4CE consortium

**DOI:** 10.1038/s41746-020-00308-0

**Published:** 2020-08-19

**Authors:** Gabriel A. Brat, Griffin M. Weber, Nils Gehlenborg, Paul Avillach, Nathan P. Palmer, Luca Chiovato, James Cimino, Lemuel R. Waitman, Gilbert S. Omenn, Alberto Malovini, Jason H. Moore, Brett K. Beaulieu-Jones, Valentina Tibollo, Shawn N. Murphy, Sehi L’ Yi, Mark S. Keller, Riccardo Bellazzi, David A. Hanauer, Arnaud Serret-Larmande, Alba Gutierrez-Sacristan, John J. Holmes, Douglas S. Bell, Kenneth D. Mandl, Robert W. Follett, Jeffrey G. Klann, Douglas A. Murad, Luigia Scudeller, Mauro Bucalo, Katie Kirchoff, Jean Craig, Jihad Obeid, Vianney Jouhet, Romain Griffier, Sebastien Cossin, Bertrand Moal, Lav P. Patel, Antonio Bellasi, Hans U. Prokosch, Detlef Kraska, Piotr Sliz, Amelia L. M. Tan, Kee Yuan Ngiam, Alberto Zambelli, Danielle L. Mowery, Emily Schiver, Batsal Devkota, Robert L. Bradford, Mohamad Daniar, Christel Daniel, Vincent Benoit, Romain Bey, Nicolas Paris, Patricia Serre, Nina Orlova, Julien Dubiel, Martin Hilka, Anne Sophie Jannot, Stephane Breant, Judith Leblanc, Nicolas Griffon, Anita Burgun, Melodie Bernaux, Arnaud Sandrin, Elisa Salamanca, Sylvie Cormont, Thomas Ganslandt, Tobias Gradinger, Julien Champ, Martin Boeker, Patricia Martel, Loic Esteve, Alexandre Gramfort, Olivier Grisel, Damien Leprovost, Thomas Moreau, Gael Varoquaux, Jill-Jênn Vie, Demian Wassermann, Arthur Mensch, Charlotte Caucheteux, Christian Haverkamp, Guillaume Lemaitre, Silvano Bosari, Ian D. Krantz, Andrew South, Tianxi Cai, Isaac S. Kohane

**Affiliations:** 1 Department of Biomedical Informatics, Harvard Medical School, Boston, MA USA; 2 IRCCS ICS Maugeri, Pavia, Italy; 3 Department of Internal Medicine and Medical Therapy, University of Pavia, Pavia, Italy; 4 UAB Informatics Institute, Birmingham, AL USA; 5 Department of Internal Medicine, Division of Medical Informatics, University of Kansas Medical Center, Kansas City, KS USA; 6 Department of Computational Medicine & Bioinformatics, University of Michigan, Ann Arbor, MI USA; 7 Institute for Biomedical Informatics, University of Pennsylvania Perelman School of Medicine, Philadelphia, PA USA; 8 Department of Biostatistics, Epidemiology, and Informatics, University of Pennsylvania Perelman School of Medicine, Philadelphia, PA USA; 9 Department of Neurology, Massachusetts General Hospital, Boston, MA USA; 10 Department of Electrical Computer and Biomedical Engineering, University of Pavia, Pavia, Italy; 11 Department of Pediatrics, University of Michigan Medical School, Ann Arbor, MI USA; 12 Department of Medicine, David Geffen School of Medicine at UCLA, Los Angeles, CA USA; 13 Computational Health Informatics Program, Boston Children’s Hospital, Boston, MA USA; 14 Department of Medicine, Massachusetts General Hospital, Boston, MA USA; 15 Scientific Direction, IRCCS Ca’ Granda Ospedale Maggiore Policlinico di Milano, Milano, Italy; 16 BIOMERIS (BIOMedical Research Informatics Solutions), Pavia, Italy; 17 Biomedical Informatics Center, Medical University of South Carolina, Charleston, SC USA; 18 Bordeaux University Hospital, Bordeaux, France; 19 UOC Ricerca, Innovazione e Brand Reputation, ASST Papa Giovanni XXIII, Bergamo, Italy; 20 Department of Medical Informatics, University of Erlangen-Nürnberg, Erlangen, Germany; 21 Center for Medical Information and Communication Technology, University Hospital Erlangen, Erlangen, Germany; 22 National University Health Systems, Singapore, Singapore; 23 Department of Oncology, ASST Papa Giovanni XXIII, Bergamo, Italy; 24 Penn Medicine, Data Analytics Center, Philadelphia, PA USA; 25 Department of Biomedical and Health Informatics, Children’s Hospital of Philadelphia, Philadelphia, PA USA; 26 North Carolina Translational and Clinical Sciences (NC TraCS) Institute, UNC Chapel Hill, Chapel Hill, NC USA; 27 WIND Department APHP Greater Paris University Hospital, Paris, France; 28 Department of Biomedical Informatics, HEGP, APHP Greater Paris University Hospital, Paris, France; 29 Clinical Research Unit, Saint Antoine Hospital, APHP Greater Paris University Hospital, Paris, France; 30 Strategy and Transformation Department, APHP Greater Paris University Hospital, Paris, France; 31 Heinrich-Lanz-Center for Digital Health, University Medicine Mannheim, Heidelberg University, Mannheim, Germany; 32 INRIA Sophia-Antipolis—ZENITH Team, LIRMM, Montpellier, France; 33 Institute of Medical Biometry and Statistics, Medical Center, University of Freiburg, Freiburg im Breisgau, Germany; 34 Clinical Research Unit, Paris Saclay, APHP Greater Paris University Hospital, Paris, France; 35 SED/SIERRA, Inria Centre de Paris, Paris, France; 36 Université Paris-Saclay, Inria, CEA, Paris, France; 37 Clevy.io, Paris, France; 38 SequeL, Inria Lille, Paris, France; 39 ENS, PSL University, Paris, France; 40 Institute of Digitalization in Medicine, Faculty of Medicine and Medical Center, University of Freiburg, Freiburg im Breisgau, Germany; 41 IRCCS Ca’ Granda Ospedale Maggiore Policlinico di Milano, Milano, Italy; 42 Brenner Children’s Hospital, Wake Forest School of Medicine, Winston-Salem, NC USA

**Keywords:** Viral infection, Outcomes research, Databases

## Abstract

We leveraged the largely untapped resource of electronic health record data to address critical clinical and epidemiological questions about Coronavirus Disease 2019 (COVID-19). To do this, we formed an international consortium (4CE) of 96 hospitals across five countries (www.covidclinical.net). Contributors utilized the Informatics for Integrating Biology and the Bedside (i2b2) or Observational Medical Outcomes Partnership (OMOP) platforms to map to a common data model. The group focused on temporal changes in key laboratory test values. Harmonized data were analyzed locally and converted to a shared aggregate form for rapid analysis and visualization of regional differences and global commonalities. Data covered 27,584 COVID-19 cases with 187,802 laboratory tests. Case counts and laboratory trajectories were concordant with existing literature. Laboratory tests at the time of diagnosis showed hospital-level differences equivalent to country-level variation across the consortium partners. Despite the limitations of decentralized data generation, we established a framework to capture the trajectory of COVID-19 disease in patients and their response to interventions.

## Introduction

The Coronavirus Disease 2019 (COVID-19) pandemic has caught the world off guard, reshaping ways of life, the economy, and healthcare delivery all over the globe. The virulence and transmissibility of responsible virus (SARS-CoV-2) is striking. Crucially, there remains a paucity of relevant clinical information to drive response at the clinical and population levels. Even in an information technology-dominated era, fundamental measurements to guide public health decision-making remain unclear. Knowledge still lags on incidence, prevalence, case-fatality rates, and clinical predictors of disease severity and outcomes. While some of the knowledge gaps relate to the need for further laboratory testing, data that should be widely available in electronic health records (EHRs) have not yet been effectively shared across clinical sites, with public health agencies, or with policy makers. At the time of this writing, more than 3 months after the earliest reports of the disease in China, only 5.8% of US cases reported to the CDC included clinical details^1^.

Even before therapeutic trials are implemented, frontline clinicians are not yet benefitting from knowledge as basic as understanding the differences in the clinical course between male and female patients^2^. Through case studies and series, we have learned that COVID-19 can have multi-organ involvement. A growing literature has identified key markers of cardiac^3^, immune^4^, coagulation^5^, muscle^5,6^, hepatic^7^, and renal^8^ injury and dysfunction, including extensive evidence of myocarditis and cardiac injury associated with severe disease. Laboratory perturbations in lactate dehydrogenase (LDH), C-reactive protein (CRP), and procalcitonin^9^ have been described. However, data from larger cohorts, linked to outcomes, remain unavailable.

Because EHRs are not themselves agile analytic platforms, we have been successfully building upon the open source and free i2b2 (for Informatics for Integrating Biology and the Bedside) toolkit^10–17^ to manage, compute, and share data extracted from EHRs. In response to COVID-19, we have organized a global community of researchers, most of whom are or have been members of the i2b2 Academic Users Group, to rapidly set up an ad hoc network that can begin to answer some of the clinical and epidemiological questions around COVID-19 through data harmonization, analytics, and visualizations. The Consortium for Clinical Characterization of COVID-19 by EHR (4CE)—pronounced “foresee”—comprises partner hospitals from five countries.

Our early efforts aim to consolidate, share, and interpret data about the clinical trajectories of the infection in patients with a first focus on laboratory values and comorbidities. This initial report seeks (a) to establish the accessibility and suitability of data from electronic medical record for COVID-19 patients; (b) to learn about the clinical trajectories of patients; (c) to facilitate evaluation and communication of the utility of various laboratory tests and therapies; and (d) to contribute data, reproducible data mining and visualization workflows, and learnings to a global network and the broader public.

Here, we report on initial results and the structure of a new, rapidly formed network designed to be a highly scalable system, now implemented at 23 sites. The international scope of our collaboration allows us to identify some of the similarities in clinical course and a few country-specific variations. We recognize that these early data are incomplete and are subject to many biases and limitations, which constrain the conclusions we can currently draw. However, we believe the sources of our data and the mechanism we have established for sharing them are sound, reproducible, and scalable. We also hope our results to-date will encourage other sites to share data and contribute to this important research effort.

## Results

### Demographic and consortium-level data

Over a span of 3 weeks, 96 total hospitals in the US (45), France (42), Italy (5), Germany (3), and Singapore (1) contributed data to the consortium. This was represented by 23 data collaboratives across these five countries. A total of 27,584 patients with COVID-19 diagnosis were included in the data set, with data covering January 1, 2020 through April 11, 2020. We collected 187,802 laboratory values and harmonized them across sites. Thirteen percent of sites submitted complete data sets that included values for each laboratory (39.1% for at least 13, and 43.5% for at least 12 of the 14 laboratory measurements). Breakdown of sites is shown in Table 1.

**Table 1:** Sites contributing data to the consortium.

Healthcare system	Acronym	City	Country	Population	Hospitals	Beds	Inpatient discharges/year
Assistance Publique—Hôpitaux de Paris	APHP	Paris	France	Adult & Pediatric	39	20,098	1,375,538
Bordeaux University Hospital	FRBDX	Bordeaux	France	Adult & Pediatric	3	2,676	130,033
Erlangen University Hospital	UKER	Erlangen	Germany	Adult & Pediatric	1	1,400	65,000
Medical Center, University of Freiburg	UKFR	Freiburg	Germany	Adult & Pediatric	1	1,660	71,500
University Medicine Mannheim	UMM	Mannheim	Germany	Adult & Pediatric	1	1,352	50,748
ICSM Pavia Hospital	ICSM1	Pavia	Italy	Adult	1	426	8616
ICSM Lumezzane/Brescia Hospitals	ICSM5	Lumezzane/Brescia	Italy	Adult	1	149	1296
ICSM Milano Hospital	ICSM20	Milan	Italy	Adult	1	200	2432
Policlinico di Milano	POLIMI	Milan	Italy	Adult & Pediatric	1	900	40,000
ASST Papa Giovanni XXIII Bergamo	HPG23	Bergamo	Italy	Adult & Pediatric	1	1080	45,000
National University Hospital	NUH	Singapore	Singapore	Adult & Pediatric	1	1556	100,977
Boston Children’s Hospital	BCH	Boston, MA	USA	Pediatric	1	404	28,000
Beth Israel Deaconess Medical Center	BIDMC	Boston, MA	USA	Adult	1	673	40,752
Children’s Hospital of Philadelphia	CHOP	Philadelphia, PA	USA	Pediatric	1	564	25,406
University of Kansas Medical Center	KUMC	Kansas City, KS	USA	Adult & Pediatric	1	794	54,659
Mayo Clinic	MAYOC	Rochester, MN	USA	Adult & Pediatric	1	2059	100,000
Mass General Brigham (Partners Healthcare)	MGB	Boston, MA	USA	Adult & Pediatric	10	3418	163,521
Medical University of South Carolina	MUSC	Charleston, SC	USA	Adult & Pediatric	8	1600	55,664
University of Pennsylvania	UPenn	Philadelphia, PA	USA	Adult	5	2469	118,188
University of California, LA	UCLA	Los Angeles, CA	USA	Adult & Pediatric	2	786	40,526
University of Michigan	UMICH	Ann Arbor, MI	USA	Adult & Pediatric	3	1000	49,008
University of North Carolina at Chapel Hill	UNC	Chapel Hill, NC	USA	Adult & Pediatric	11	3095	52000
UT Southwestern Medical Center	UTSW	Dallas, TX	USA	Adult	1	608	26,905
				Total	96	45,352	2,444,792

**Fig. 1:**
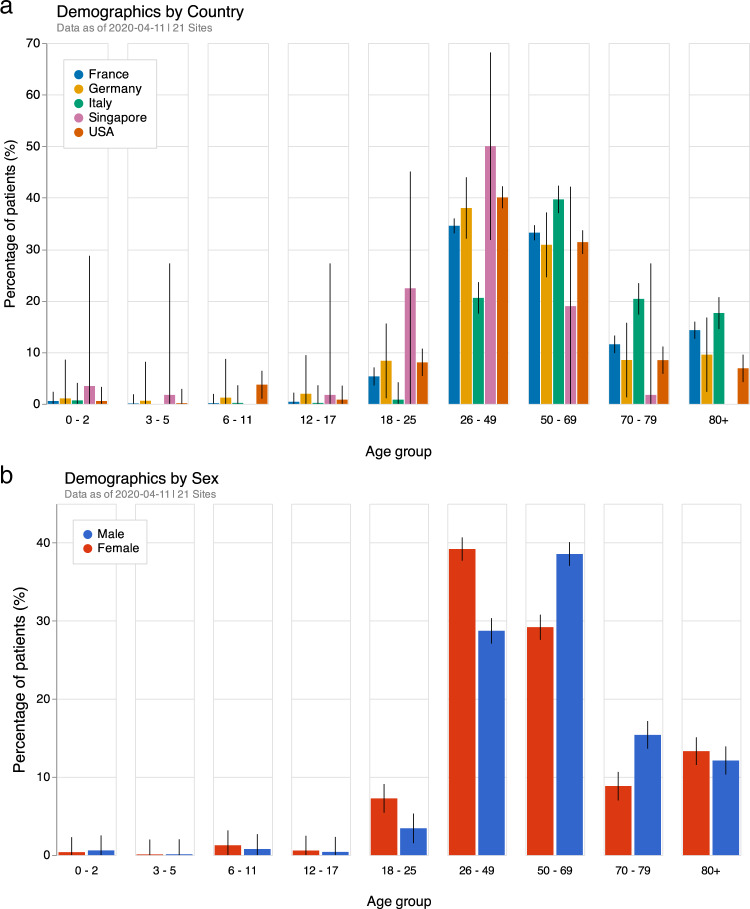
Percentages of patients along with 95% confidence intervals in each **a** country and **b** sex age groups.

Demographic breakdown by age and sex is shown in Fig. 1. Age distribution was different across countries and consistent with previously identified patterns. In particular, patients from Italy were more commonly over the age of 70 relative to other countries^18^. US institutions, despite representing a large number of active infections, had the lowest percentage of elderly patients diagnosed with COVID-19. Germany, with its three included hospitals and relatively small number of patients, was more similar to the US and had an increased number of male patients in the 50−59 age group.

**Fig. 2:**
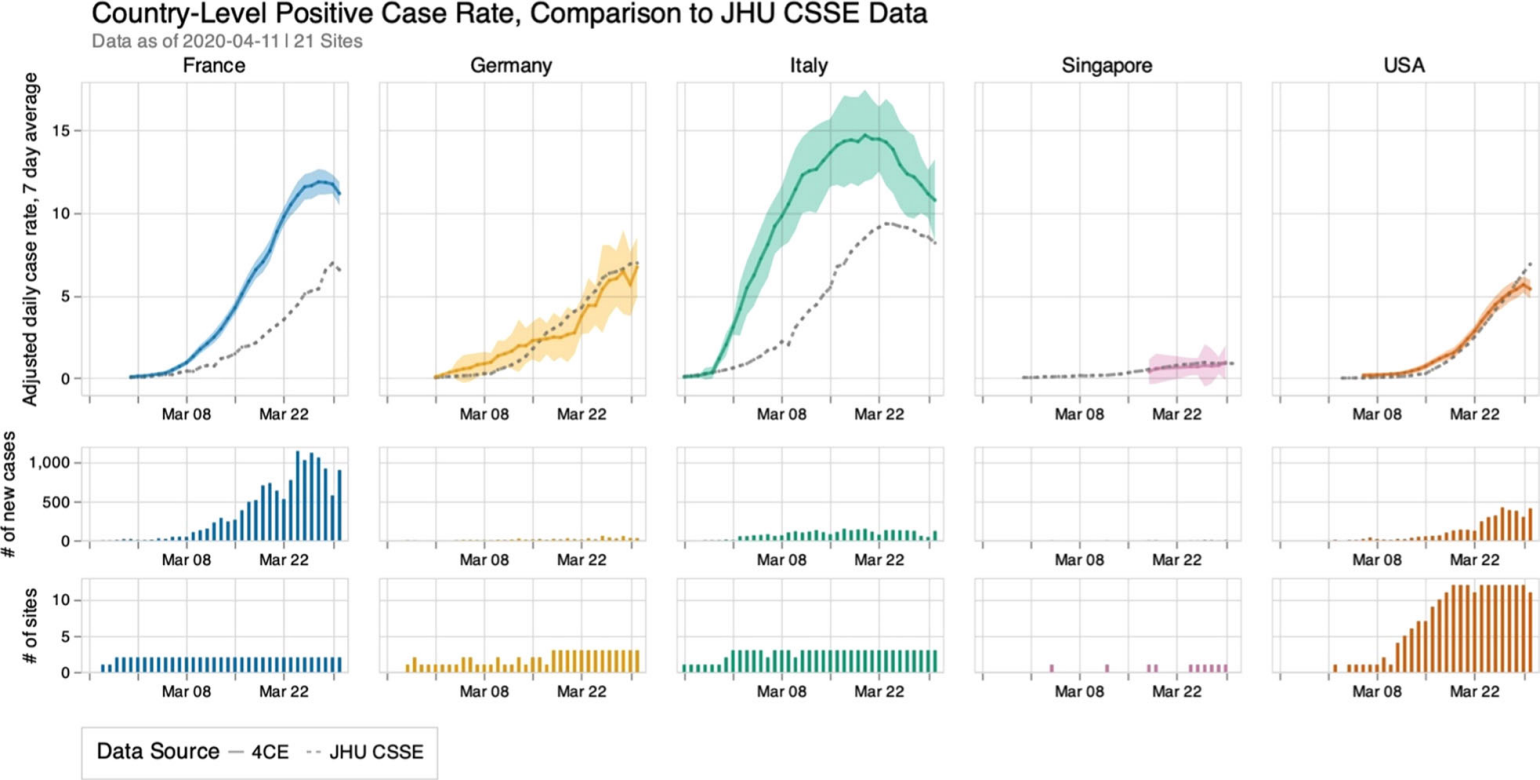
Adjusted 7-day average new case rate per 100K by country based on 4CE contributors along with 95% confidence intervals compared to 7-day average new case rate collected by Johns Hopkins Center for Systems Science and Engineering (JHU CSSE).

We were able to capture the total number of identified new cases by site and date. To normalize across sites and countries with varying sizes, we reported 7-day average new case rate per 100K over time for each country normalized by the ratio between the inpatient discharge rate for each country and inpatient discharge rate for the 4CE sites in that country. As shown in Fig. 2, the adjusted 7-day average new case rates derived from 4CE consortium sites match reasonably well with those reported by JHU CSSE^19^ for Germany, US, and Singapore. The 4CE estimates were substantially higher for France and Italy, which could reflect the fact that 4CE sites in France and Italy were mainly concentrated in urban areas with high infection rates.

### Laboratory value trajectories

**Fig. 3:**
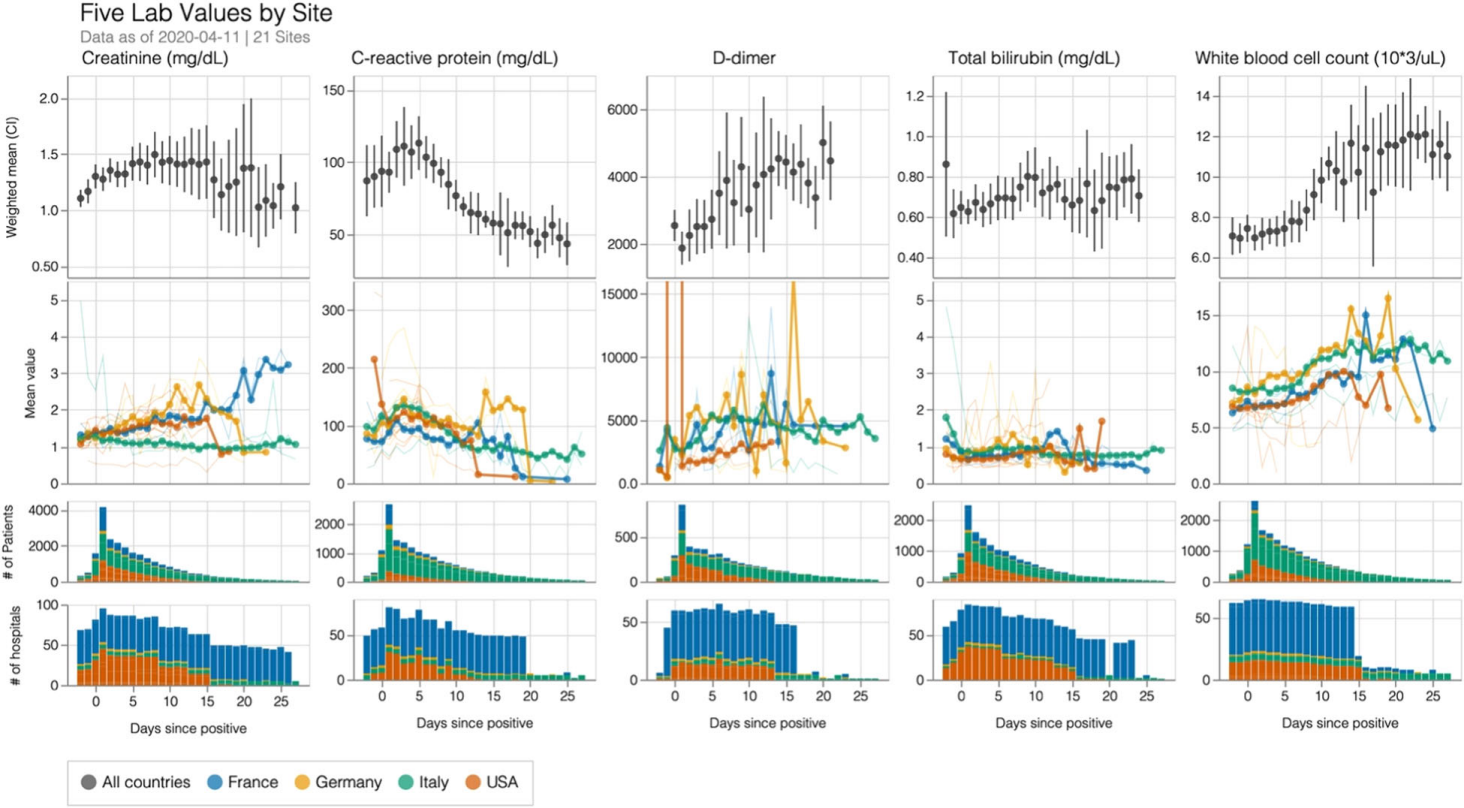
Laboratory tests representative of renal function (creatinine), systemic inflammation (C-reactive protein), coagulopathy (D-dimer), liver function (total bilirubin), and immune response (white blood cell count) visualized relative to date of diagnosis of COVID-19. The top row shows weighted means and 95% confidence intervals across all patients. The second row shows unweighted country- (thick lines) and site-level (thin lines) means. The third and fourth rows show the number of patients and sites, respectively, contributing laboratory tests of each type on a given day.

Our initial data extraction included 14 laboratory markers of cardiac, renal, hepatic, and immune dysfunction that have been strongly associated with poor outcomes in COVID-19 patients in previous publications. Laboratory trajectories of each hospital at the population level are presented online at https://covidclinical.net. Given limitations of data harmonization and space, we focused on five laboratory trajectories that represented inflammatory, immune, hepatic, coagulation, and renal function. As shown in Fig. 3, trajectory data were remarkably consistent for most institutions at day 1 (day when biological test was positive) with growing differences with continued hospitalization. Extensive data harmonization was performed, but we must emphasize that data from each day represented a potentially different population as patients were discharged, died, or laboratory studies were no longer performed. Data values from each hospital were an average of all studied patients a specified number of days after diagnosis.

Initial laboratory values were abnormal for all patients but were not indicative of organ failure. Major abnormal elevations were noted in CRP and D-dimer on the day of diagnosis. As the number of days from diagnosis progressed, remaining patients who were not discharged or died had, on average, worse values. For nearly all 14 tests, trends toward progressively abnormal values were consistent with worsening disease as inpatient stays continued. Most importantly, the initial values and trajectories were highly consistent with previous findings in studies from China^20,21^.

Creatinine, a measure of renal function and the most commonly performed laboratory test in our data set, was divergent over time across sites. Rising creatinine would be consistent with an increased proportion of ill patients with significant acute kidney injury over time. Hospitals in Italy, in contrast, did not see a dramatic rise in creatinine in their hospitalized population, while the small percentage of French and German patients remaining in the hospital for 2 weeks had clear signs of acute kidney injury. This may represent many underlying differences including a high mortality near the beginning of the hospitalization at Italian hospitals, severe right time censoring of remaining patients, or a difference in practice.

Total bilirubin, a measure of conjugation and function by the liver, was initially normal across most sites and showed increases—consistent with other hepatic laboratory tests—among persistently hospitalized patients. The other hepatic laboratory measurements, alanine aminotransferase (ALT) and aspartate aminotransferase (AST), were divergent across institutions and showed a more significant perturbation (see https://covidclinical.net). Hepatic impairment was not present in most patients on presentation and total bilirubin was only mildly elevated with continued hospitalization.

On average, white blood cell count (WBC), a measure of immune response, was within normal limits on presentation. Patients who remained in the hospital and survived had increasing WBCs over time without severe leukocytosis^21^. Lymphocyte and neutrophil count trajectories can be seen on the website. Procalcitonin and LDH were not commonly tested in the total patient population, but results are also online.

C-reactive protein, a measure of systemic inflammation, was notably elevated on presentation for all patients in the cohort with a very narrow confidence interval, consistent with previous findings^20^. Although it is of unclear importance, populations of patients who remained in the hospital, survived, and had ongoing laboratory testing showed improvements over time. Interestingly, despite a decreasing trajectory during the first week, a mild leukocytosis is observed in counterbalance during the second week. The implication may be that CRP is not predictive of ongoing hospitalization or CRP is being checked for patient populations where the laboratory is more commonly improving.

D-dimer, an acute phase reactant and measure of coagulopathy, was elevated across institutions and countries at presentation. It rose consistently in all populations who continued to be hospitalized with the disease. This was consistent with multiple studies that showed a prothrombotic element to the disease. Most importantly, changes were consistent across all sites and highly abnormal.

### Data attrition

**Fig. 4:**
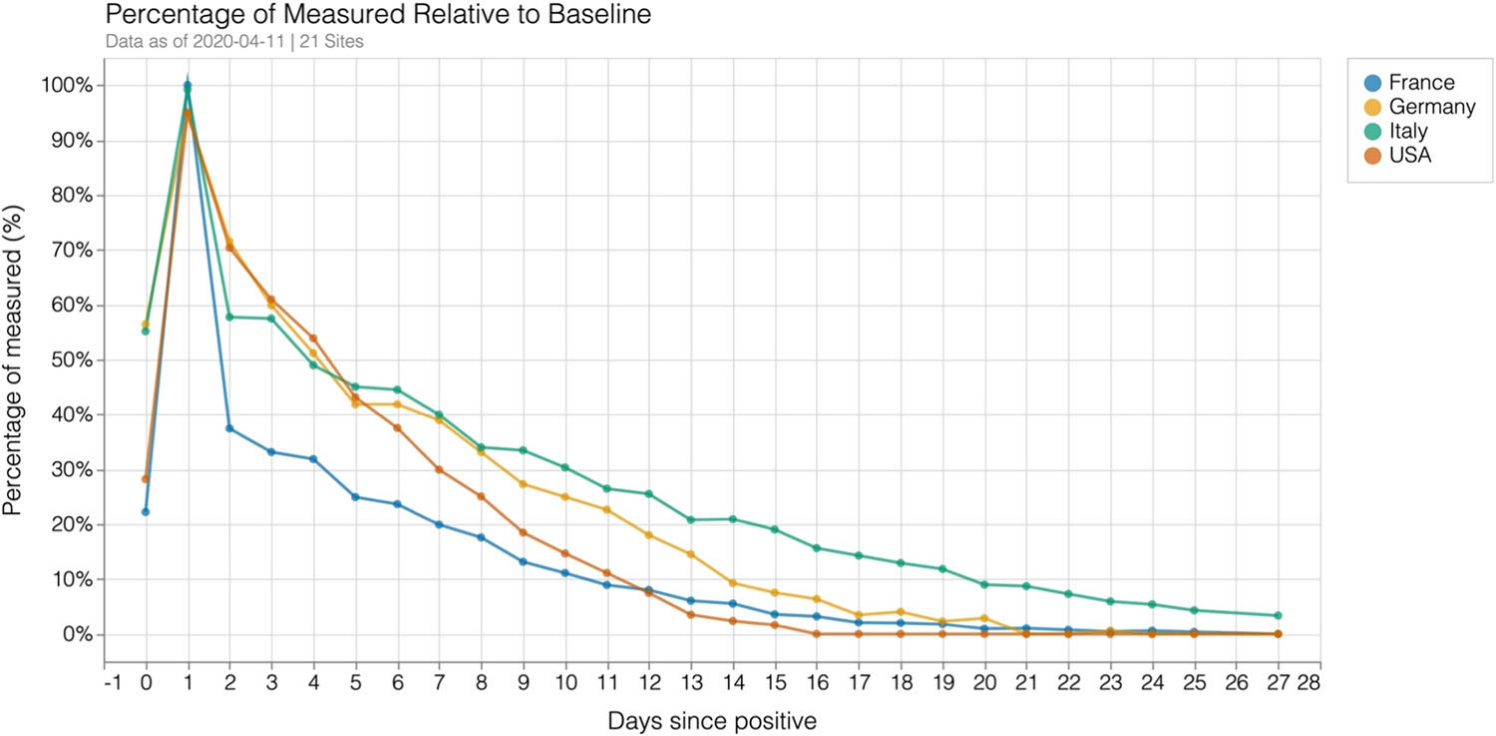
Drop-off in laboratory tests reported for creatinine relative to the day with the largest number of laboratory tests reported.

There was a large drop in the number of laboratory tests performed after the first day (see Fig. 4). Drop off in tests performed could be a result of death, length of stay, or change in frequency of data collection by the clinical team. From the maximum number of laboratory tests consistently checked on the first day after diagnosis, there was a rapid tapering in frequency of laboratory tests checked. These changes were particularly pronounced in Italy and France. We identified the number of days until the number of tests checked were 20% of their initial maximum value. Values for laboratory study for each day are presented on https://covidclinical.net. Results varied for each laboratory value and site. There was no obvious country-level pattern. Given that several of these tests, such as creatinine, were commonly checked nearly every day in ill patients, the implication was that patients were censored from the laboratory results because of discharge or death or changing practice pattern. Thus, for the purposes of this paper, we focused on trends in creatinine. We normalized the number of tests performed by day to the total performed on day 1. We then looked at the day when the number of tests performed was 20% of the maximum number performed for each site. For creatinine, for example, a drop-off in testing occurred between day 7 and 15 across institutions. Most patients who survived were likely discharged within this time frame or managed with much less monitoring. Further results can be found online.

### Differences at admission

**Fig. 5:**
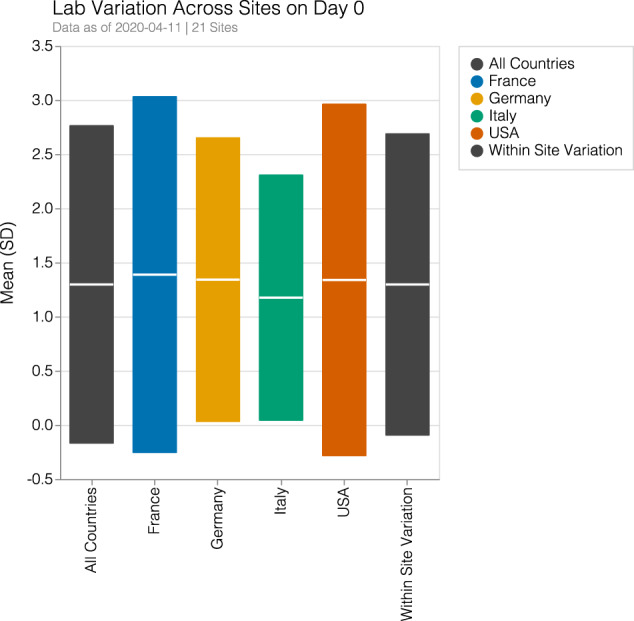
Laboratory variation across countries and within sites for creatinine test performed within a day of diagnosis. Values for mean value and standard deviation (SD) in creatinine level are shown. Large variations exist within sites and are often larger than the between country variation. The overall SD across countries was 1.47 while the SD within sites was 1.39. Standard deviation for countries was 1.64, 1.31, 1.13, and 1.62 within France, Germany, Italy, and the US, respectively.

There was greater between-hospital variation for laboratory test performance than between-country variation (see Fig. 5). At the time of diagnosis, there was significant variation between countries and between the hospitals in a specific country. There was no obvious signature presentation for a country for an individual laboratory value. For example, creatinine was a commonly performed laboratory study within a day of diagnosis. The overall standard deviation (SD) for creatinine values across countries was 1.47 while the SD within sites was 1.39. Standard deviation for countries was 1.64, 1.31, 1.13, and 1.62 within France, Germany, Italy, and the US, respectively. France was a special case as 39 hospitals were reported together by AP-HP and then compared with three hospitals in Bordeaux. This was an important finding that could suggest that laboratory values, as individual results, would not be able to fully explain the mortality differences between countries.

## Discussion

A rapid mobilization of a multi-national consortium was able to harmonize and integrate data across five countries and three continents in order to begin to answer questions about comparative care of COVID-19 patients and opportunities for international learning. In just over 2 weeks, the group was able to define a question and data model, perform data extraction and harmonization, evaluate the data, and create a site for public evaluation of site-level data. We aggregated EHR data from 96 hospitals, covering a total of 27,584 patients seen in these hospitals for COVID-19. In doing so, we relied upon prior investments made by various governments and institutions in turning the byproducts of clinical documentation into data useful for a variety of operational and scientific tasks. Most importantly, at each site there were biomedical informatics experts who understood both the technical characteristics of the data and their clinical relevance.

Using automated data extraction methods, we were able to show results consistent with country-level demographic and epidemiological differences identified in the literature. Rates of total case rise in our study was consistent with international tracking sites^19^. Age breakdown, with Italian sites reporting a larger proportion of older patients, was also reflective of recent publicly available resources^18^.

We were able to show that laboratory trajectories across many hospitals could be collected and were concordant with findings from the literature. In truth, the findings generate more questions than they answer; the ability to see consistencies that spanned many countries indicated that the pathophysiology of this disease is shared across countries, and that demographics and care characteristics will have a significant effect on outcomes. As an example, the fall of CRP among those who continued to be hospitalized with a continued rise in d-dimer could suggest that d-dimer may be more closely related to persistent illness than CRP. The limits of our data collection method, where these results were not tied to the patient level and could not be associated across populations, highlight the need for caution with any conclusion related to changes in laboratory levels over time.

Perhaps most importantly, our study did not show a unique laboratory signature at the country level at the time of diagnosis. Researchers around the world have been closely following the rapid spread of COVID-19 and its high mortality rate in certain countries. One possible explanation would be that patients who presented to hospitals in Italy did so at a much more advanced stage of disease. Our results did not support this idea. There was as much in-hospital and between-hospital variation as between countries.

The average of laboratory values at presentation did not indicate major organ failure. This may be due to a larger proportion of healthier patients than those with advanced disease. Of course, respiratory failure could not be tracked within the limits of our data set.

There were both logistic and data interoperability lessons that were very important to the success of the project and will be critical for future efforts. Logistically, to maximize the timeliness of this consortium’s first collaboration around COVID-19, we deliberately aggregated the data to expedite the institutional review board (IRB) process at each institution for such data sharing. This constrained our analyses to count, rather than patient-level, data. While the latter would be optimal for deep analysis and identification of subtle patterns and perturbations of clinical courses, we felt that aggregated count data could provide valuable information on the clinical course even as we sought IRB permission for analyses at the patient level.

Interoperability was a significant barrier to overcome, where large variations in units and data presentation required extensive data harmonization. The use of LOINC codes allowed for more rapid data extraction^22–25^, but often institutions did not have internal mappings from their laboratory tests to LOINC codes. Manual interpretation of laboratory value descriptions was sometimes necessary. In future iterations, sites should perform unit conversion and ensure data consistency by presenting reference ranges and example data for a first-pass check of data at the site. Variations in ICD coding and inclusion made harmonization difficult. Frequencies of presenting codes were useful to show similar patterns to previous literature, but the current set of codes was too sparse for any further meaningful analysis. Future iterations of this project would encompass a much longer data capture timeline and would ensure comprehensive code collection across all sites.

In addition, data alignment by a metric that indicates clinical status is necessary to better establish outcomes. Using day of diagnosis as an alignment strategy did not allow for clear identification of causes for temporal patterns. This was, in part, because we could not differentiate between patients who underwent lab testing and were not admitted. Although additional lab testing was performed almost exclusively for admitted patients, it is possible that some emergency department patients were triaged and sent home. This would explain the rapid drop-off (and subsequent leveling) seen after day 1 in Fig. 4. Future studies will need to explicitly differentiate between categories of patients admitted and triaged to home. These care choices may not reflect similar patient physiology but will more readily track care provision. Similarly, outcomes need to be selected that represent clinically meaningful endpoints secondary to this initial data alignment. One reason for this difficulty was that identification of level of care was not easily performed. Accordingly, it was not easy to follow patients in and out of ICUs at the site-level and ICU data were not reliable.

Our group, the Consortium for Clinical Characterization of COVID-19 by EHR (4CE), is one of hundreds of efforts (some of which are listed at HealthIT.gov) that are working to aggregate and curate data to inform clinicians, scientists, policy makers, and the general public. Additionally, networks of healthcare organizations such as the ACT network^26^ and PCORnet^27^ are working with federal authorities to obtain data-driven population-level insights. Similar initiatives are active in the other countries participating in 4CE, including the German Medical Informatics Initiative^28^. Disease-specific and organ-specific COVID-19 research collectives are also assembling, including ones for cancers (https://ccc19.org), inflammatory bowel disease (https://covidibd.org), and rheumatology^29^, among many others. The World Health Organization maintains a directory of worldwide research efforts on COVID-19 including clinical data collection^30^. Finally, there are dozens of patient self-reporting apps with hundreds of thousands of users worldwide that provide perspectives on the clinical course of the infection outside hospitals.

It is clear that in the midst of a novel pathogen, uncertainty far outstrips knowledge. At this early stage, we are partially blind to the underlying physiology of the disease and its interactions with different health system processes. The rapid collation of laboratory-level data across nearly 100 hospitals in five countries is novel in the questions it helps us ask. We are currently struggling to help public health agencies and hospitals better manage the epidemic. By identifying potential differences in care, with proxies of lab changes over time, numerous questions can be asked about whether certain clinical decisions may be affecting lab trajectories (and ultimately outcomes). As an example, differences in creatinine over time may be a signal of patient-level physiology or hospital decisions about care. The regional clustering of the trajectories identified is striking and deserves further analysis. Could there be choices in diuresis and fluid management that may explain differing trajectories? If so, best practice may need to change to the specific physiology of this disease. We have been treating COVID-19 like previous infections despite its unique physiology; with the right information, our scientific and policy leaders can implement guidelines that improve care.

There are a multitude of limitations to this study, not least of which is that it is observational and subject to a variety of biases. Perhaps the most severe is that study data are limited to those patients who were seen at or admitted to hospitals, due to severity of illness or other possibly biasing characteristics. Aggregate laboratory data have limited ability to identify general trends in the admitted population. Changes in the cohort as a result of discharge or death may change the composition of the cohort over time. The time-varying average represents the labs of remaining patients in the hospital; survivors who require ongoing care. This leads to a survivor bias. Because there is significant patient drop-out, the remaining population cannot be compared to the initial cohort. Our study is only able to identify that patients had similar initial labs suggesting consistent initial physiology. It is not possible to use these values as drivers of outcomes such as death or severe disease. Differences in health capacity may also lead to differences in admitted patients that ultimately manifest as worse outcomes across institutions or countries. Limitations also include heavy right censoring where patient absence can be due to death or discharge, delays in updating codes or in uploading EHR data to the local analytic data repository. Furthermore, potentially confounding interactions between comorbidities, chronic diseases and their treatments and lifestyle or exposures were not taken into consideration. Again, because of these limitations we were careful to avoid making more than basic and descriptive conclusions. Over the coming weeks, we will work to quantify these biases and adjust for them, if we can. This will include adding data types as well as disaggregating the data to the patient level if and when permitted by IRBs. For the present, with the current limited knowledge of the clinical course of patients suffering from COVID-19, these results add to this small knowledge base. Our paper strikingly shows the power of harmonized data extraction from EHRs to rapidly study pandemics like COVID-19. By example, we hope we can motivate an international discussion on what would be required to enable such international monitoring to simply and rapidly be turned on in future COVID-19 “waves” or in future novel pandemics.

We invite others to join the 4CE consortium by sending a note to 4CE@i2b2foundation.org.

## Methods

### Selection of laboratory values

Multiple studies have reported significant abnormalities in several laboratory tests in patients with COVID-19. Studies have shown abnormalities in cardiac, hepatic, renal, immune, and coagulation physiology. Those laboratory results are associated with both disease presentation and severity. For this initial study, we selected a subset of laboratory studies that are commonly performed, as identified by the Logical Objects, Identifiers, Names and Codes (LOINC) standard^31^, and had been previously associated with worse outcomes in COVID-19 patients. Based on the meta-analysis of Lippi and Plebani^21^, we focused on 14 laboratory studies that are commonly performed: ALT, AST, total bilirubin (Tbili), albumin, cardiac troponin (high sensitivity), LDH, D-dimer, white blood cell count (WBC), lymphocyte count, neutrophil count, procalcitonin, and prothrombin time. LOINC codes were identified for each laboratory study as well as the units and reference ranges.

### Cohort identification

All patients who received a polymerase chain reaction (PCR)-confirmed diagnosis of COVID-19 were included in the data collection. Some hospitals only included patients who were admitted to the hospital while others included all patients for whom the test was positive.

### Data collection and aggregation

Sites obtained the data for their files in several ways. Most sites leveraged the open source i2b2 software platform already installed at their institution^32^, which supports query and analysis of clinical and genomics data. More than 200 organizations worldwide use i2b2 for a variety of purposes, including identifying patients for clinical trials, drug safety monitoring, and epidemiology research. Most 4CE sites with i2b2 used database scripts to directly query their i2b2 repository to calculate counts needed for data files. Institutions without i2b2 used their own clinical data warehouse solutions and querying tools to create the files. In some cases, a hybrid method was used that leveraged different data warehouse platforms to fill in i2b2 gaps. For example, Assistance Publique—Hôpitaux de Paris (APHP), the largest hospital system in Europe, aggregates all EHR data from 39 hospitals in Paris and its surroundings. APHP exported data from the Observational Medical Outcomes Partnership (OMOP) Common Data Model for transformation to the shared format.

Each site generated four data tables, saved as comma-separated values (CSV) files. To protect patient privacy, the files we report contain only aggregate counts (no data on individual patients). In order to further protect patient identity, small counts were obfuscated (see below), since an aggregate count of “1” represents an individual patient. By computing these values locally and only sharing the aggregate data, sites were able to obtain institutional approval more rapidly.

The first file, *DailyCounts.csv*, contained one row per calendar date. Each row included the date, the number of new COVID-19 patients, the number of COVID-19 patients in an intensive care unit (ICU), and the number of new deaths from COVID-19.

The second file, *Demographics.csv*, contained counts of the total number of COVID-19 patients, broken down by sex and age group (0−2, 3−5, 6−11, 12−17, 18−25, 26−49, 50−69, 70−79, and 80+ years old).

The third file, *Labs.csv*, described the daily trajectories of select laboratory tests. Each row corresponded to a laboratory test (identified using a LOINC code) and the number of days since a patient had a positive COVID-19 test, ranging from −6 (1 week before the test result) to 1 (the day of the test result) to *N* (the day the file was created). The values in each row were the number of patients who had a test result on that day and the mean and standard deviation of the test results.

The fourth file, *Diagnoses.csv*, listed all the diagnoses recorded in the EHR for COVID-19 patients, starting from 1 week before their positive COVID-19 test to the present, with the count of the number of patients with the corresponding ICD-9 or ICD-10 code.

Sites optionally obfuscated the values in any of these files by replacing small counts with “−1.” Sites indicated missing data or data that they were unable to obtain (e.g. whether patients were in an ICU) with “−2.”

Sites uploaded their files to a private shared folder. These files were merged into four combined files that included totals from individual sites. Each value in the combined file had four components: (1) number of sites with unmasked values; (2) sum of those values; (3) number of sites with obfuscated values; and (4) sum of the obfuscation thresholds for those sites. For example, if five sites reported values 25, 15, −1 (between 00 and 9 patients), −1 (between 00 and 4 patients), −1 (between 00 and 4 patients), then the combined file listed two unmasked sites with a total of 40 patients and three masked sites with up to 9 +4 + 4 = 17 patients. From this, it was inferred that there were between 40 and 57 patients. Given the large geographic distance between our sites, we assumed that each COVID-19 patient was only represented in one EHR. The combined *Labs.csv* file contained a weighted average (rather than the sum) of the unmasked mean test results from each site.

### ICD mapping

Diagnosis codes were submitted from the sites as either international clinical diagnosis (ICD)-9 or ICD-10 billing codes. ICD-9 diagnosis codes were mapped to ICD-10 by first attempting to match the ICD-9 codes to child concepts of ICD-10 codes in the Accrual to Clinical Trials (ACT) ICD-10 → ICD-9 ontology^33^. In the cases where no match was found in the ACT ontology, ICD-9 codes were matched to the ICD-10 codes that shared a common concept unique identifier (CUI) in the 2019 build of the US National Library of Medicine’s (NLM’s) Unified Medical Language System (UMLS)^34^.

### Data sharing and visualization

**Fig. 6:**
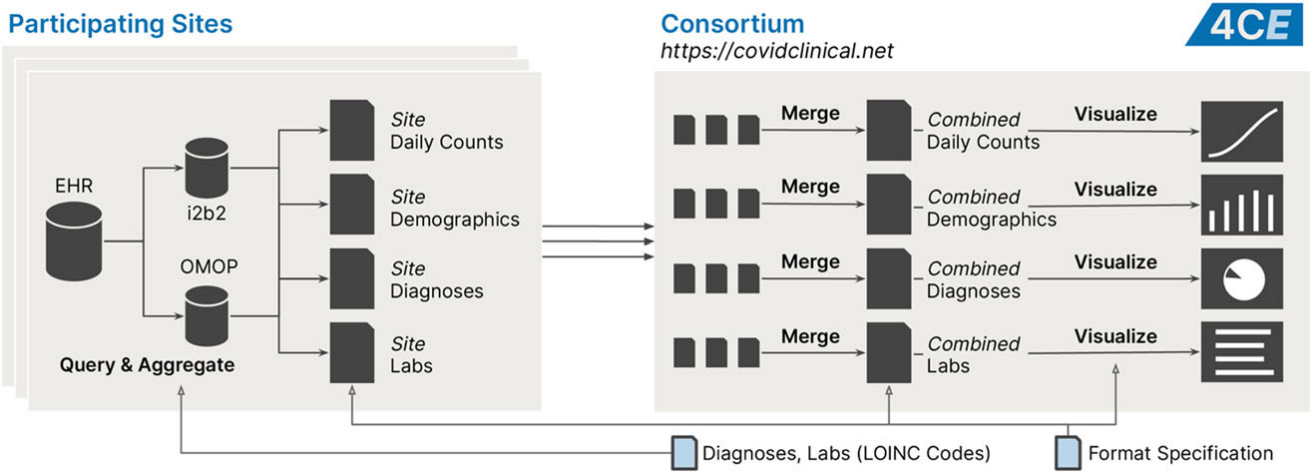
Overview of data collection and analysis.

We created a website hosted at https://covidclinical.net to provide interactive visualizations of our data sets as well as direct access to all shareable data collected for this publication. Data aggregation and publication processes are shown in Fig. 6. Visualizations were implemented using Python and Altair (http://altair-viz.github.io/) in Jupyter Notebooks (https://jupyter.org), all of which are freely available on the website. The Vega visualizations (http://vega.github.io) generated by Altair were embedded into a Jekyll-based site (http://jekyllrb.com/) that was hosted on Amazon Web Services.

### Informed consent/IRB statement

This study was determined to be exempt as secondary research by the Partner’s HealthCare, Boston Children’s Hospital and Beth Israel Deaconess Medical Center. The committee collected certifications of proper institutional review board prior to data sharing for each additional member of the consortium. As data were transmitted in aggregate, no patient-level data were available from any site.

### Reporting summary

Further information on experimental design is available in the Nature Research Reporting Summary linked to this article.

## Supplementary information

Supplementary Material

Reporting Summary

## Data Availability

Data files for daily counts, demographics, diagnosis, and labs data sets are available at https://covidclinical.net.
