# Supplementary material for: International electronic health record-derived COVID-19 clinical course profiles: the 4CE consortium
Source: NPJ Digit Med. 2020 Aug 19;3:109. doi: 10.1038/s41746-020-00308-0 (PMC7438496; doi:10.1038/s41746-020-00308-0)
Supplement: Supplementary file 1 — Supplementary Material [file 41746_2020_308_MOESM1_ESM.pdf]

# Supplementary Material

## Author Contributions

| **First Name** | **Last Name** | Conception or design of the work | Acquisition of data | Analysis or Interpretation of data | Drafting Manuscript | Approval |
|---|---|---|---|---|---|---|
| Isaac | Kohane | x | x | x | x | x |
| Tianxi | Cai | x | x | x | x | x |
| Gabriel | Brat | x | x | x | x | x |
| Paul | Avillach | x | x | x | x | x |
| Griffin | Weber | x | x | x | x | x |
| Kenneth | Mandl | x | x | | x | x |
| Nathan | Palmer | x | | x | x | x |
| Riccardo | Bellazzi | x | x | | x | x |
| Luca | Chiovato | | x | | x | x |
| Valentina | Tibollo | | x | x | x | x |
| Alberto | Malovini | | x | x | x | x |
| Lav | Patel | | x | x | x | x |
| Lemuel | Waitman | | x | x | x | x |
| Shawn | Murphy | | | x | x | x |
| Jeffrey | Klann | | x | x | x | x |
| Nils | Gehlenborg | x | | x | x | x |
| Sehi | L'Yi | | | x | x | x |
| Mark | Keller | | | x | x | x |
| Gilbert | Omenn | x | | x | x | x |
| David | Hanauer | | x | x | x | x |
| Arnaud | Serret-Larmande | | | x | x | x |
| Alba | Gutiérrez-Sacristán | | | x | x | x |
| John | Holmes | | | x | x | x |
| Douglas | Bell | | | x | x | x |

| | | | | | | |
|---|---|---|---|---|---|---|
| Robert | Follett | | | x | x | x |
| Douglas | Murad | | | x | x | x |
| Luigia | Scudeller | | | x | x | x |
| Mauro | Bucalo | | x | x | x | x |
| Katie | Kirchoff | | x | | x | x |
| Jean | Craig | | x | | x | x |
| Jihad | Obeid | | x | | x | x |
| Vianney | Jouhet | | x | | x | x |
| Romain | Griffier | | x | x | x | x |
| Sébastien | Cossin | | x | x | x | x |
| Bertrand | Moal | | x | x | x | x |
| Hans | Prokosch | | x | | x | x |
| Detlef | Kraska | | x | | x | x |
| Piotr | Sliz | | x | | x | x |
| Amelia | Tan | | x | x | x | x |
| Kee Yuan | Ngiam | | x | x | x | x |
| Antonio | Bellasi | | x | | x | x |
| Alberto | Zambelli | | x | | x | x |
| Brett | Beaulieu-Jones | | x | x | x | x |
| Danielle | Mowery | | x | | x | x |
| Emily | Schiver | | x | | x | x |
| Jason | Moore | | x | x | x | x |
| Batsal | Devkota | | x | | x | x |
| Robert | Bradford | | x | x | x | x |
| Mohamad | Daniar | | x | | x | x |
| Christel | Daniel | | x | x | x | x |
| Vincent | Benoit | | x | | x | x |
| Romain | Bey | | x | | x | x |
| Nicolas | Paris | | x | x | x | x |
| Patricia | Serre | | x | x | x | x |
| Nina | Orlova | | x | x | x | x |

| | | | | | | |
|---|---|---|---|---|---|---|
| Julien | Dubiel | | x | | x | x |
| Martin | Hilka | | x | | x | x |
| Anne Sophie | Jannot | | x | x | x | x |
| Stéphane | Bréant | | x | | x | x |
| Judith | Leblanc | | x | | x | x |
| Nicolas | Griffon | | x | | x | x |
| Anita | Burgun | | x | x | x | x |
| Mélodie | Bernaux | | x | x | x | x |
| Arnaud | Sandrin | | x | x | x | x |
| Elisa | Salamanca | | x | x | x | x |
| Sylvie | Cormont | | x | x | x | x |
| Thomas | Ganslandt | | x | | x | x |
| Tobias | Gradinger | | x | | x | x |
| Julien | Champ | | x | x | x | x |
| Martin | Boeker | | x | | x | x |
| Patricia | Martel | | x | x | x | x |
| Loic | Esteve | | x | x | x | x |
| Alexandre | Gramfort | | x | x | x | x |
| Olivier | Grisel | | x | x | x | x |
| Guillaume | Lemaitre | | x | x | x | x |
| Damien | Leprovost | | x | x | x | x |
| Thomas | Moreau | | x | x | x | x |
| Gael | Varoquaux | | x | x | x | x |
| Jill Jen | Vie | | x | x | x | x |
| Demian | Wassermann | | x | x | x | x |
| Arthur | Mensch | | x | x | x | x |
| Charlotte | Caucheteux | | x | x | x | x |
| Christian | Haverkamp | | x | x | x | x |
| James | Cimino | | | x | x | x |
| Ian | Krantz | | x | | x | x |
