# Supplementary material for: International electronic health record-derived COVID-19 clinical course profiles: the 4CE consortium
Source: NPJ Digit Med. 2020 Aug 19;3:109. doi: 10.1038/s41746-020-00308-0 (PMC7438496; doi:10.1038/s41746-020-00308-0)
Supplement: Supplementary file 2 — Reporting Summary [file 41746_2020_308_MOESM2_ESM.pdf]

nature research

Corresponding author(s): Tianxi Cai and Isaac Kohane

Last updated by author(s): May 20, 2020

Reporting Summary

Nature Research wishes to improve the reproducibility of the work that we publish. This form provides structure for consistency and transparency in reporting. For further information on Nature Research policies, see our Editorial Policies and the Editorial Policy Checklist.

Statistics

For all statistical analyses, confirm that the following items are present in the figure legend, table legend, main text, or Methods section.

n/a	Confirmed	
☐	☒	The exact sample size (n) for each experimental group/condition, given as a discrete number and unit of measurement
☐	☒	A statement on whether measurements were taken from distinct samples or whether the same sample was measured repeatedly
☐	☒	The statistical test(s) used AND whether they are one- or two-sided<br>*Only common tests should be described solely by name; describe more complex techniques in the Methods section.*
☐	☒	A description of all covariates tested
☐	☒	A description of any assumptions or corrections, such as tests of normality and adjustment for multiple comparisons
☐	☒	A full description of the statistical parameters including central tendency (e.g. means) or other basic estimates (e.g. regression coefficient) AND variation (e.g. standard deviation) or associated estimates of uncertainty (e.g. confidence intervals)
☐	☒	For null hypothesis testing, the test statistic (e.g. F, t, r) with confidence intervals, effect sizes, degrees of freedom and P value noted<br>*Give P values as exact values whenever suitable.*
☒	☐	For Bayesian analysis, information on the choice of priors and Markov chain Monte Carlo settings
☐	☒	For hierarchical and complex designs, identification of the appropriate level for tests and full reporting of outcomes
☐	☒	Estimates of effect sizes (e.g. Cohen's d, Pearson's r), indicating how they were calculated

Our web collection on statistics for biologists contains articles on many of the points above.

Software and code

Policy information about availability of computer code

Data collection	Aggregate data were submitted by each participating site using either i2b2 or OMOP representations - https://transmartfoundation.org/platforms/. Most 4CE sites with i2b2 used database scripts to directly query their i2b2 repository to calculate counts needed for data files. Institutions without i2b2 used their own clinical data warehouse solutions and querying tools to create the files. In some cases, a hybrid method was used that leveraged different data warehouse platforms to fill in i2b2 gaps. For example, Assistance Publique – Hôpitaux de Paris (APHP), the largest hospital system in Europe, aggregates all EHR data from 39 hospitals in Paris and its surroundings. APHP exported data from the Observational Medical Outcomes Partnership (OMOP) Common Data Model for transformation to the shared format.
Data analysis	https://github.com/covidclinical/visualization-notebooks

For manuscripts utilizing custom algorithms or software that are central to the research but not yet described in published literature, software must be made available to editors and reviewers. We strongly encourage code deposition in a community repository (e.g. GitHub). See the Nature Research guidelines for submitting code & software for further information.

Data

Policy information about availability of data

All manuscripts must include a data availability statement. This statement should provide the following information, where applicable:

- Accession codes, unique identifiers, or web links for publicly available datasets
- A list of figures that have associated raw data
- A description of any restrictions on data availability

All Data available at: https://covidclinical.net/data/index.html and in archival repositories: https://doi.org/10.6084/m9.figshare.12152967.v1, https://doi.org/10.6084/m9.figshare.12118911.v3

Field-specific reporting

Please select the one below that is the best fit for your research. If you are not sure, read the appropriate sections before making your selection.

☒ Life sciences ☐ Behavioural & social sciences ☐ Ecological, evolutionary & environmental sciences

For a reference copy of the document with all sections, see nature.com/documents/nr-reporting-summary-flat.pdf

Life sciences study design

All studies must disclose on these points even when the disclosure is negative.

Sample size	All patients who received a polymerase chain reaction (PCR) confirmed diagnosis of COVID-19 were included in the data collection.
Data exclusions	NA
Replication	This is a multi-site, international study with thorough comparison across 23 institutions and 96 hospitals to measure statistical variability.
Randomization	Randomization was not possible and is not relevant because this study does not attempt to estimate an intervention effect size or match between populations.
Blinding	Blinding was not possible and is not relevant to this observational profile of the COVID-19 Clinical Course.

Reporting for specific materials, systems and methods

We require information from authors about some types of materials, experimental systems and methods used in many studies. Here, indicate whether each material, system or method listed is relevant to your study. If you are not sure if a list item applies to your research, read the appropriate section before selecting a response.

Materials & experimental systems

n/a	Involved in the study	
☒	☐	Antibodies
☒	☐	Eukaryotic cell lines
☒	☐	Palaeontology and archaeology
☒	☐	Animals and other organisms
☒	☐	Human research participants
☐	☒	Clinical data
☒	☐	Dual use research of concern

Methods

n/a	Involved in the study	
☒	☐	ChIP-seq
☒	☐	Flow cytometry
☒	☐	MRI-based neuroimaging

Clinical data

Policy information about clinical studies

All manuscripts should comply with the ICMJE guidelines for publication of clinical research and a completed CONSORT checklist must be included with all submissions.

Clinical trial registration	NA
Study protocol	Study Methods Section.
Data collection	Over a span of three weeks, 96 total hospitals in the US (45), France (42), Italy (5), Germany (3), and Singapore (1) contributed data to the consortium. This was represented by 23 data collaboratives across these five countries. A total of 27,584 patients with COVID-19 diagnosis were included in the dataset, with data covering January 1, 2020 through April 11, 2020. We collected 187,802 laboratory values and harmonized them across sites.

Outcomes

We examined key lab value trajectories (Creatinine, Total bilirubin, white blood cell count, C-reactive protein, D-dimer) in order to profile the clinical course of COVID-19.
